# Audiovisual interaction with rate-varying signals

**DOI:** 10.1177/20416695221116653

**Published:** 2022-11-28

**Authors:** Long Yi, Robert Sekuler

**Affiliations:** Volen Center for Complex Systems, 8244 Brandeis University, Waltham, MA, USA

**Keywords:** audiovisual interaction, confusion matrix, similarity space, temporal correlation, rate perception

## Abstract

A task-irrelevant, amplitude-modulating sound influences perception of a size-modulating visual stimulus. To probe the limits of this audiovisual interaction we vary the second temporal derivative of object size and of sound amplitude. In the study’s first phase subjects see a visual stimulus size-modulating with $f^{\prime \prime }(x)>$0, 0, or <0, and judge each one’s rate as increasing, constant, or decreasing. Visual stimuli are accompanied by a steady, non-modulated auditory stimulus. The novel combination of multiple stimuli and multi-alternative responses allows subjects’ similarity space to be estimated from the stimulus-response confusion matrix. In the study’s second phase, rate-varying visual stimuli are presented in concert with auditory stimuli whose second derivative also varied. Subjects identified each visual stimuli as one of the three types, while trying to ignore the accompanying sound. Unlike some previous results with $f^{\prime \prime }(x)$ fixed at 0, performance benefits relatively little when visual and auditory stimuli share the same directional change in modulation. However, performance does drop when visual and auditory stimului differ in their directions of rate change. Our task’s computational demands may make it particularly vulnerable to the effects of a dynamic task-irrelevant stimulus.

The combination of signals from multiple sensory modalities has attracted interest from a variety of researchers, including neuroscientists (Andric et al., 2017; Peelle & Sommers, 2015), sensory researchers (Payne & Sekuler, 2014; Spence, 2011), and computer scientists working on automated video annotation (Cavaco et al., 2012). Signal combination has been of particular interest for research on speech intelligibility. It has long been known that a conversation’s intelligibility is enhanced when a listener can see the movements of a speaker’s face. This particular beneficial interaction between seeing and hearing, called audiovisual speech, can be especially important in noisy environments like crowded restaurants and parties (Peelle & Sommers, 2015). The many complexities associated with speech stimuli and with measures of intelligibility (Lam et al., 2017; Reetzke et al., 2021) encouraged researchers to develop low-dimensional putative analogues of audiovisual speech (Maddox et al., 2015).

A set of recent studies of audiovisual interaction presented subjects with a visual stimulus, for example, a disc, whose size could modulate sinusoidally at either of two different rates. Subjects made binary judgments of modulation rate, categorizing what they saw as either the slower or faster rate (Goldberg et al., 2015; Sun & Sekuler, 2021; Sun et al., 2017; Tai et al., 2022; Varghese et al., 2017). On some occasions, the size-modulating disc was accompanied by a concurrent, amplitude-modulating sound that subjects were told to ignore. Visual modulation rate was more accurately categorized when visual and auditory stimuli shared the same modulation frequency than when they modulated at different rates. In these studies, modulation rate was fixed throughout the entire stimulus presentation. That temporal constancy differs from the complex dynamics of many natural events, such as animal locomotion and human speech, where multiple factors influence pace. Also, dynamics have long been exploited for a variety of musical effects. Examples from Western popular music include Queen’s “Bohemian Rhapsody” and Taylor Swift’s “evermore”; examples from Western classical music can be found in Mozart’s “Dissonant” String Quartet, and Brahms’ 4th Hungarian Dance.

To examine how temporal variation impacts the combination of auditory and visual inputs, we introduced low-dimensional dynamics into both the task-relevant visual stimulus (a white disc whose size varied sinusoidally), and into the task-irrelevant auditory stimulus (a tone whose amplitude modulated sinusoidally). These temporal dynamics parallel ones that have been studied with auditory and somatosensory stimuli whose temporal frequencies sweep (change monotonically) over time. For example, while exploring the organization of spectral processing in sensory systems, Crommett et al. (2019) found that auditory frequency sweeps altered the perception of tactile frequency sweep. Several studies have exploited visual stimuli whose spatial frequency is swept over time (Norcia & Tyler, 1985; Tyler et al., 1979), but we believe ours is the first in which the temporal frequency of visual stimuli is swept.

To increase our study’s potential information yield, we expanded the task’s decision-making framework away from binary choices to one entailing multiple alternatives (Nunes & Gurney, 2016; Tajima et al., 2019). This change in the task is consequential because the information needed for binary decisions is relatively simple compared to the sensory information needed for multi-alternative decisions (Tsetsos et al., 2011; Yeon & Rahnev, 2020). We took advantage of expanded multi-alternative stimulus-response contingencies to gain insight into subjects’ perceptual similarity space (Edelman & Shahbazi, 2012; Shepard, 1987).

## Method

**Figure 1.**
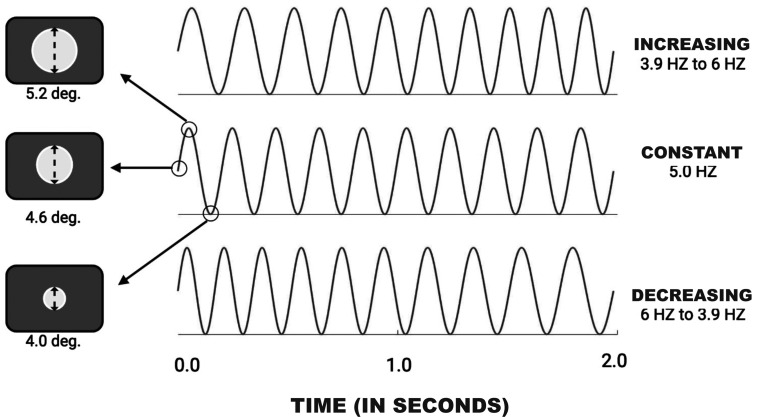
Three types of frequency sweep for modulation rate: linearly increasing rate, constant rate, and linearly decreasing rate. The same modulation changes were applied to both auditory and visual stimuli, and same modulation changes were used in Phases A and B of the study. The three diagrams to the left of the waveforms represent the disc’s size at the maximum, middle, and minimum of the sinusoidal modulation. Note that the disc diameter, 4.6${}^{o}$ visual angle, was always the same at the start and end of a trial, eliminating size as a potential cue to stimulus type. In Phase A of our study, a visual stimulus could modulate in any of the three fashions shown in the diagram, while the accompanying steady auditory stimulus was unmodulated; in Phase B, any of the three possible types of visual modulation were crossed with amplitude-modulated auditory stimuli whose rates of modulation varied in the same three ways.

Our study comprised two phases, which were run as a series of interleaved, alternating blocks of trials. Throughout, subjects saw a white disc whose size modulated with $f^{\prime \prime }(x)>$0, 0, or <0, and judged its rate as increasing, constant, or decreasing. Phase A of the study produced control, baseline measures for judgments of the visual stimulus, and allowed us to examine the perceptual similarity structure of those stimuli; Phase B explored the effect of combining visual and auditory stimuli whose modulation rates varied over time. In order to minimize possible differential effects of variables such as practice and fatigue, the two phases were alternated over eight blocks of 50 trials each. On approximately equal numbers of random trials in either phase, the disc’s size modulation rate could (i) slowly increase, (ii) remain constant at 5 Hz, or (iii) slowly decrease. Figure 1 illustrates the three stimulus types schematically. When modulation rate varied during a stimulus presentation, the variation for both visual and auditory stimuli was linear, at 21% s${}^{-1}$ and both had modulation depths of 13%. Subjects performed the same task in both phases, namely, categorizing the change in the disc’s size modulation rate as increasing, constant, or decreasing. Blocks of trials were separated by short rest breaks.

The disc in Phase A was accompanied by a steady amplitude. Results from this phase provided measures of baseline sensitivity to change in visual modulation rate, unperturbed by variation in the concurrent sound. Phase B presented the same visual stimuli as in Phase A, but now the accompanying tone modulated in ampltude, and at a rate that linearly increased or decreased in frequency, at 21% sec${}^{-1}$, or remained constant (at 5 Hz). Note that we will use the term “Steady” to describe Phase A’s unmodulated concurrent sound, and the term “Constant” to refer to Phase B’s *f*’(x) = 0 accompanying auditory stimulus.

### Subjects

Subjects were Brandeis University undergraduates who participated for partial course credit. As this experiment was closely related to previous experiments that had 13–29 subjects (Goldberg et al., 2015; Sun & Sekuler, 2021; Sun et al., 2017; Tai et al., 2022; Varghese et al., 2017), we collected data from 26 subjects, a number toward the upper end of related experiments’ range. Experimental procedures were approved by Brandeis University’s Institutional Review Board and were conducted in accordance with the Declaration of Helsinki. All subjects gave written informed consent prior to participation.

### Phase A

#### Apparatus and Stimuli

The flow of the experiment, stimulus delivery, and response collection were controlled by PsychoPy (Peirce et al., 2019) running on the Pavlovia server (https://pavlovia.org/). Because of the COVID-19 pandemic, subjects were tested remotely, using their own computers and web browsers. Subjects were encouraged to use headphones or earbuds, but were free to rely on their computer’s speakers if they preferred. Prior to testing, an interactive “virtual chin-rest” routine scaled the displayed stimuli to compensate for differences in screen size, resolution, and viewing distance (Li et al., 2020; Morys-Carter, 2020). To produce sound levels that were at least roughly comparable across subjects, subjects set the loudness of the tone that would be used in the experiment to a comfortable level. To verify that subjects kept their device’s audio turned on, the auditory stimulus was muted on ∼2% of trials. On such probe trials subjects had to press the space bar to confirm that they detected the sound’s absence.

Subjects observed and than categorized the dynamics of a white disc presented at the center of a gray screen. As suggested by the diagrams in Figure 1, the disc modulated in size sinusoidally at a frequency that linearly increased, remained constant, or decreased over each two-second presentation. Compared to abrupt temporal change, perception of gradual temporal change has been shown to be relatively challenging (Gottsdanker, 1956; Gottsdanker et al., 1961; López-Moliner & Soto-Faraco, 2007; McAuley & Henry, 2010; Mueller & Timney, 2016; Watamaniuk & Heinen, 2003; Werkhoven et al., 1992), and requires a relatively long integration time (Gori et al., 2013). Preliminary testing showed when the modulation rate of our stimuli increased or decreased linearly at 21% sec${}^{-1}$, they were discriminable at levels appreciably above chance but below the upper limits of our accuracy measure. So we used that rate of change for all rate-changing auditory and visual stimuli.

In both phases of the experiment, auditory stimuli were 440 Hz tones coterminous with the presentation of the disc. Before the start of a stimulus, a fixation point guided the subjects’ attention to the screen’s center. To encourage them to attend to the stimulus over its entire duration, subjects were not permitted to respond until a stimulus had ended. They then had up to two seconds to respond, pressing one of three computer keys to signal their judgment that the disc’s size modulation rate increased, remained constant, or decreased. Because subjects were not permitted to respond until the stimulus timed-out, we could not tell when they had actually made their decision. Unfortunately, this negated the informational value of response times.

Immediately after a response, text on the screen told the subject which stimulus had been presented and whether their response had been correct. The text remained visible for one-second. Then a two-second inter-trial interval elapsed before the onset of the next trial (Sussman et al., 2021). The order in which the three stimulus types (visual modulation rate Increasing, Decreasing, or remaining Constant) were presented was randomized over trials.

### Conditions

#### Phase A

In Phase A, the three different forms of disc size modulation were accompanied by a constant amplitude 440 Hz tone that was coterminous with the presentation of the disc. Subjects were instructed to base their categorization responses on the behavior of the visual stimulus, ignoring the accompanying sound.

#### Phase B

Conditions, task, and stimuli were as in Phase A with the following exceptions. In Phase B, on some trials the 440 Hz tone that accompanied the size-modulating disc was amplitude modulated. Specifically, the tone could either modulate in amplitude at a constant frequency of 5 Hz, or modulate at a rate that increased or decreased. Specifically, when the tone’s amplitude modulation frequency was to increase, it increased linearly from 3.9 Hz at onset to 6 Hz at its end; when the modulation frequency was to decrease, it started at 6 Hz and linearly decreased to 3.9 at the end. Note that these three conditions of auditory amplitude modulation corresponded to the three types of visual size modulation (see Figure 1). As in Phase A, subjects were instructed to respond rapidly, basing their categorization judgments solely on the behavior of the disc, while ignoring the accompanying sound.

Crossing the three types of visual modulation and the three types of auditory modulation produced nine distinct combinations of auditory and visual stimuli, which were presented in random order. Note that in three of the combinations, visual and auditory stimuli shared the same type of change in modulation frequency: auditory and visual both increasing, both decreasing, or both remaining constant. These three conditions can be described as Congruent; in the remaining six conditions, auditory and visual frequency changes were Incongruent.

## Results

Data downloaded from the Pavlovia server were first checked for missed responses. Subjects who missed responding in time or failed to respond correctly to sound-absent, probe trials one standard deviation above the mean were excluded from data analysis. This criterion excluded five subjects’ data, leaving 21 subjects’ data for analysis. Subjects failed to respond in time on only 1% of sound-absent, probe trials, and on just 0.3% of non-probe trials. Those trials were not included in our analyses.

### Phase A

**Figure 2.**
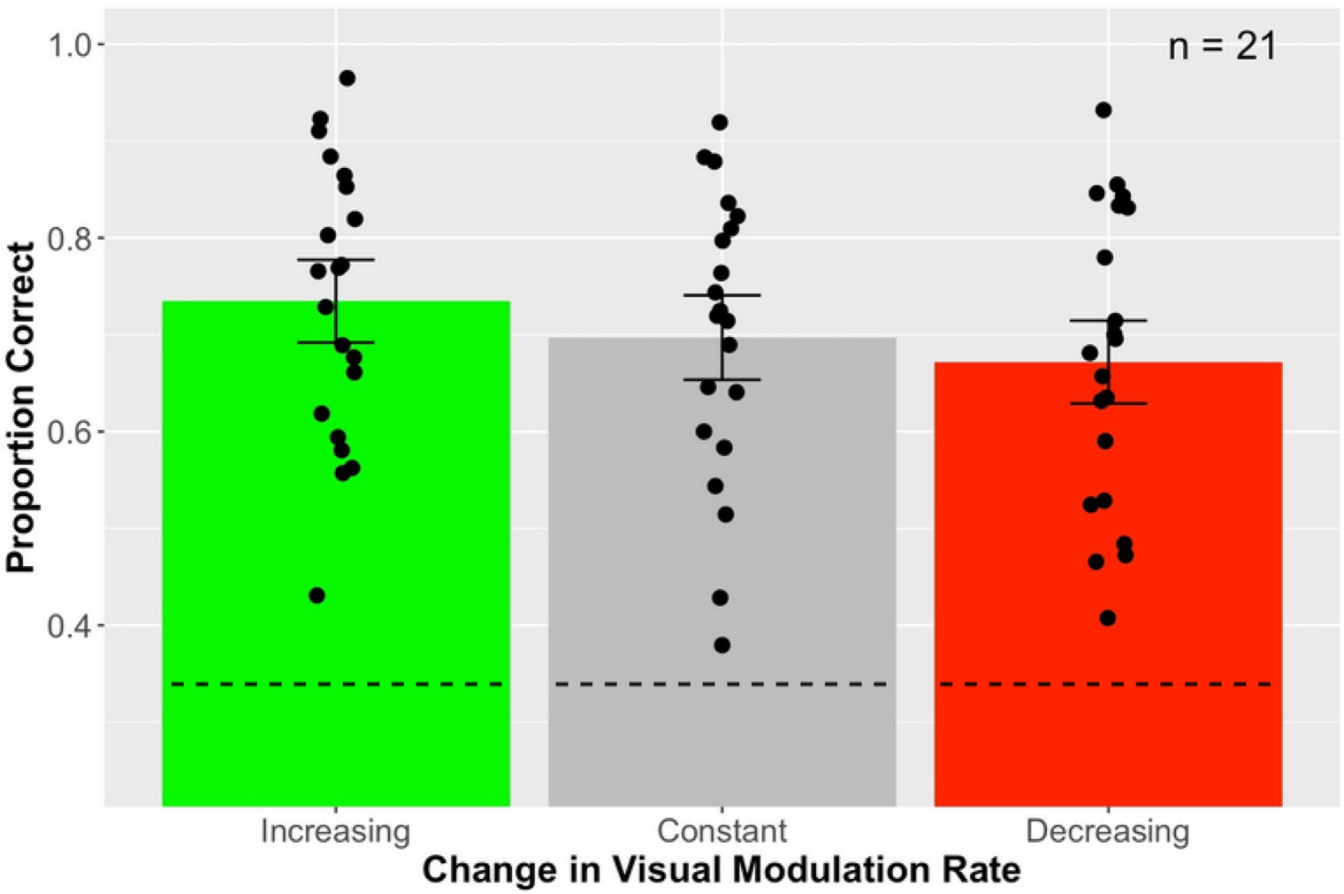
Phase A data showing response accuracy for the three types of visual modulation. Error bars span 95% within-subject confidence limits. The dashed horizontal line inside each bar represents chance performance, 0.33.

Figure 2 shows the mean accuracy in categorizing the visual modulation on which each of the three types of visual stimuli, for example, the proportion of trials on which an Increasing rate stimulus was identified as increasing. Data points represent individual subjects. Error bars show the associated 95% confidence limits around the mean for each condition. Had subjects merely guessed at random, accuracy would have been at chance level, 0.33, which is shown by the dashed horizontal lines toward the bars’ bottoms. Each of three types of visual stimuli was correctly identified at a level well above chance: *t* = 12.96, 11.33, and 10.25, for Increasing, Constant, and Decreasing stimuli, respectively, all *df*=20 and *p*<.0001. Further, a one-way repeated-measures ANOVA showed that identification accuracy did not differ reliably across the three stimulus types, *F*(2, 39.97) = 2.35, *p* = .108, $\eta_{p}^{2}$ = 0.105. All pairwise comparisons on conditions were likewise non-significant, all *p*>.14, with the Holm-Bonferroni correction for multiple comparisons. To translate mean accuracy values into signal detection terms, we can treat the task as *m*-interval forced identification, with *m*=3 (Macmillan & Creelman, 2005). That approach produces $d^{\prime }$ values of 1.35, 1.24, and.1.13, for Increasing, Constant, and Decreasing, respectively. So, however the result is expressed, subjects readily identify which type of stimulus they are seeing.

**Figure 3.**
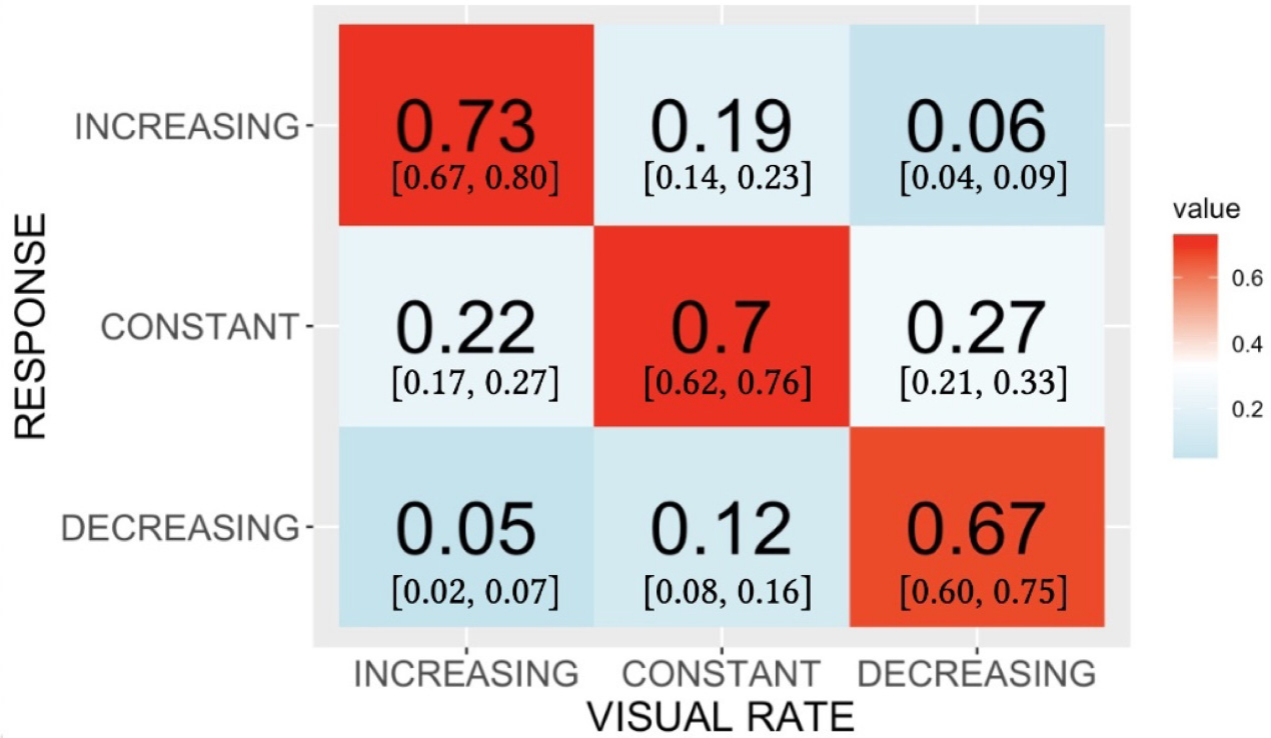
Phase A data showing the timulus-response confusion matrix with responses of the three types of visual stimuli. Values shown are proportions of responses assigned to each cell in the matrix; 95% confidence limits are given in brackets. Entries have been normalized so that the proportions within a column sum to 1.0.

The accuracy data shown in Figure 2 were produced by treating each trial’s response as binary, that is, as either correct or not. That treatment discards potentially important information contained in the way that wrong responses are distributed across the three response categories. Specifically, this distribution across stimulus-response combinations can provide clues to the perceptual similarity relationships among stimuli. To make use of this potential information, we cast the judgments made to each stimulus type into a stimulus-response confusion matrix, and converted the frequencies into normalized proportions (Landis & Koch, 1977; Simske, 2019). Figure 3 shows the result. To summarize subjects’ overall success as classifiers of the stimuli, we calculated Cohen’s K for the confusion matrix. The value obtained, 0.53, fell about halfway between the value for perfect, error-free classification (K=1.0) and the value for completely random responses (K=0).

As a way to circumscribe the relationships among the perceptual states produced by the three types of stimuli we extracted key qualitative relationships from the confusion matrix in Figure 3. First, Increasing and Decreasing stimuli were rarely mistaken for one another, that is, on just 0.08 and 0.06 of trials on which they were presented. This suggests that, unsurprisingly, of all the perceptual states represented in the matrix, those produced by Increasing and by Decreasing stimuli are most distinct from one another. Next, although all three types of stimuli were presented equally often, one response category, “constant,” drew the most incorrect responses from other types of stimuli. Moreover, Increasing stimuli and Decreasing stimuli were nearly equally likely to be mis-categorized as “constant”, 0.22 and 0.27, respectively. If perceptual responses to our visual stimuli were arrayed along a single dimension, the relationships in the confusion matrix constrain the three distributions of perceptual responses to be approximately equidistant from one another, with the distribution elicited by the Constant stimulus at the center.

### Phase B

**Figure 4.**
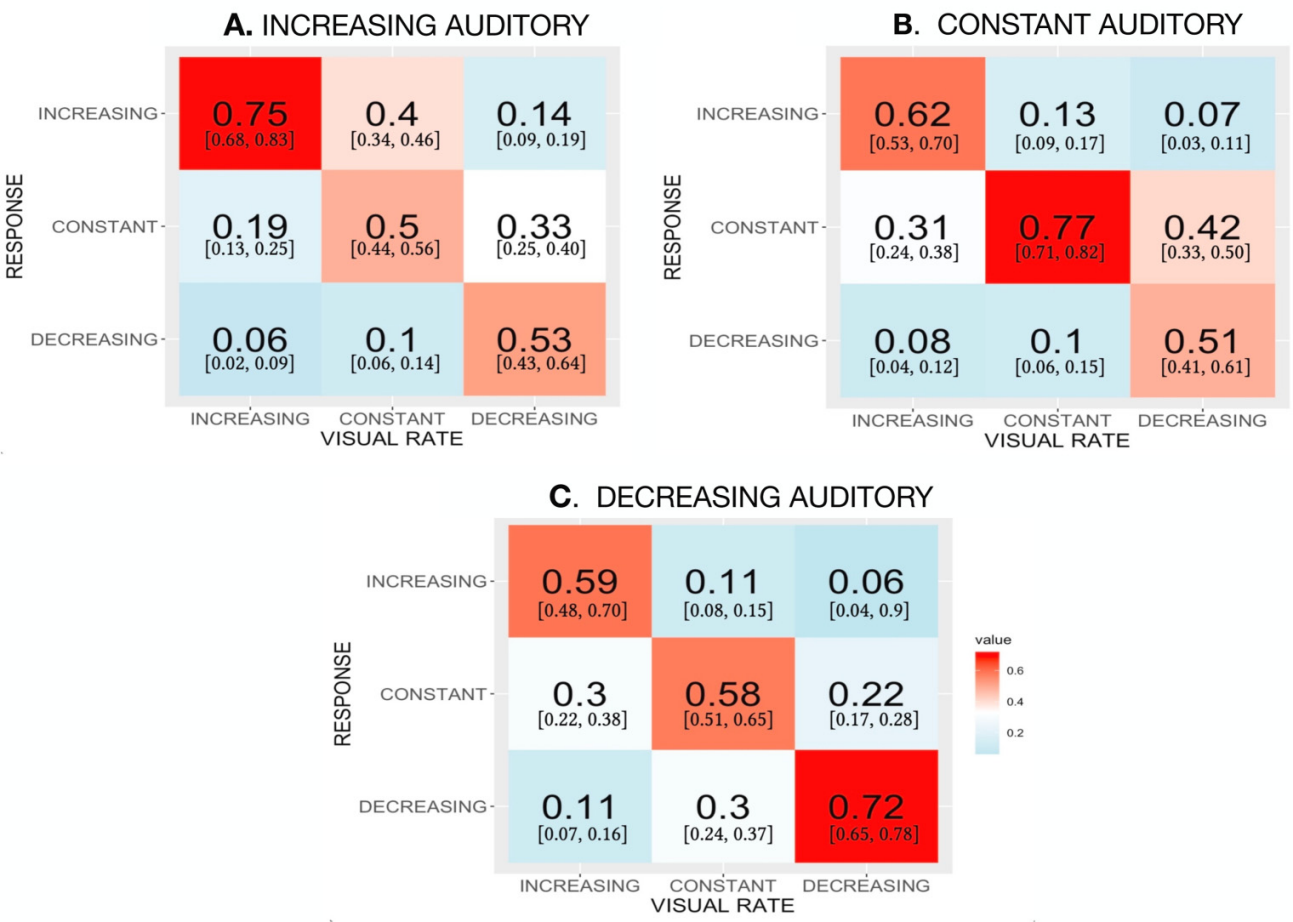
Phase B data showing the proportion of identifications assigned to each response category for each type of visual stimulus. The panels show results from different accompanying, task-irrelevant auditory stimuli: Panel A with concurrent increasing rate auditory stimuli; Panel B for auditory stimuli whose modulation rate was constant; and Panel C with concurrent decreasing rate auditory stimuli. In each cell of the panels, 95% within-subject confidence limits are given in brackets.

The three confusion matrices in Figure 4A to C show mean accuracy for identifying the rate change of visual stimuli when they are accompanied by an auditory stimulus of Increasing rate (Panel A), Constant rate (Panel B), or Decreasing rate (Panel C). In each confusion matrix, accuracy is highest when the auditory and the visual rate changes matched one another.

Individual subjects’ proportion correct responses were entered into a two-way within-subject ANOVA in which one factor was the type of visual rate change and the other factor was the type of auditory rate change. Table 1 summarizes the ANOVA’s results. Although type of visual rate change did not have a statistically significant effect (*p* = .15), the type of task-irrelevant auditory rate change did (*p* = .04). Importantly, the interaction between type of visual rate change and type of auditory rate change was significant (*p*<.001) and accounted for a substantial potion of the variance in response accuracy ($\eta_{\;p}^{2}$ = 0.614). The origin of the interaction can be understood by comparing the three confusion matrices shown in Figure 4: correct identifications of the visual stimulus type seem to shift depending upon the type of concurrent auditory stimulus (Table 2).

**Table 1.** Results of ANOVA on Phase B results.

Effect	*dfs*	*F*	*p*	$\eta_{p}^{2}$
Visual rate	1.96, 39.19	1.99	.15	0.090
Auditory rate	1.99, 39.89	3.60	.04	0.152
Interaction	3.07, 61.30	31.77	.001	0.614

Corrected for multiple comparisons using Holm-Bonferroni method.

**Table 2.** Responses on trials with the disc modulating at a constant rate.

		Sound	
		Increasing	Decreasing
Response	“Increasing”	188	50
	“Decreasing”	48	133

In a classic study of audiovisual interaction, Shipley (1964) demonstrated what he called “auditory driving,” that is, changing the rate of auditory clicks altered the perception of visual flashes presented at comparable, low rates. Later, Welch et al. (1986) confirmed this basic result with more subjects and different psychophysical method. Although the stimuli in those two studies differed from our sweep frequency stimuli, key results in Figure 4 point to auditory driving in our experiment. Shipley’s psychophysical method produced continuous, quantitative estimates of perceived visual rate following every change in auditory rate. Despite the relatively coarse grain afforded by just three alternative responses, auditory driving can be seen by comparing judgments of Constant rate visual stimuli when accompanied by an Increasing rather than a Decreasing rate auditory stimulus. In fact, Figure 4 offers evidence of auditory driving. Specifically, when Constant visual stimuli are misidentified, those misidentifications tend toward the direction in which the auditory rate changed. Specifically, with an Increasing auditory rate (Figure 4A), the Constant visual stimulus is misidentified as “increasing” on 0.40 [0.34, 0.46] of trials, but misidentified as “decreasing” on only 0.10 [0.06, 0.14] of trials. The result with a Decreasing rate auditory stimulus (Figure 4C) is the opposite, namely, Constant visual stimulus are pulled toward “decreasing” judgments on 0.30 [0.24, 0.37] of trials, but toward “increasing” on only 0.11 [0.08, 0.15] of trials.

**Figure 5.**
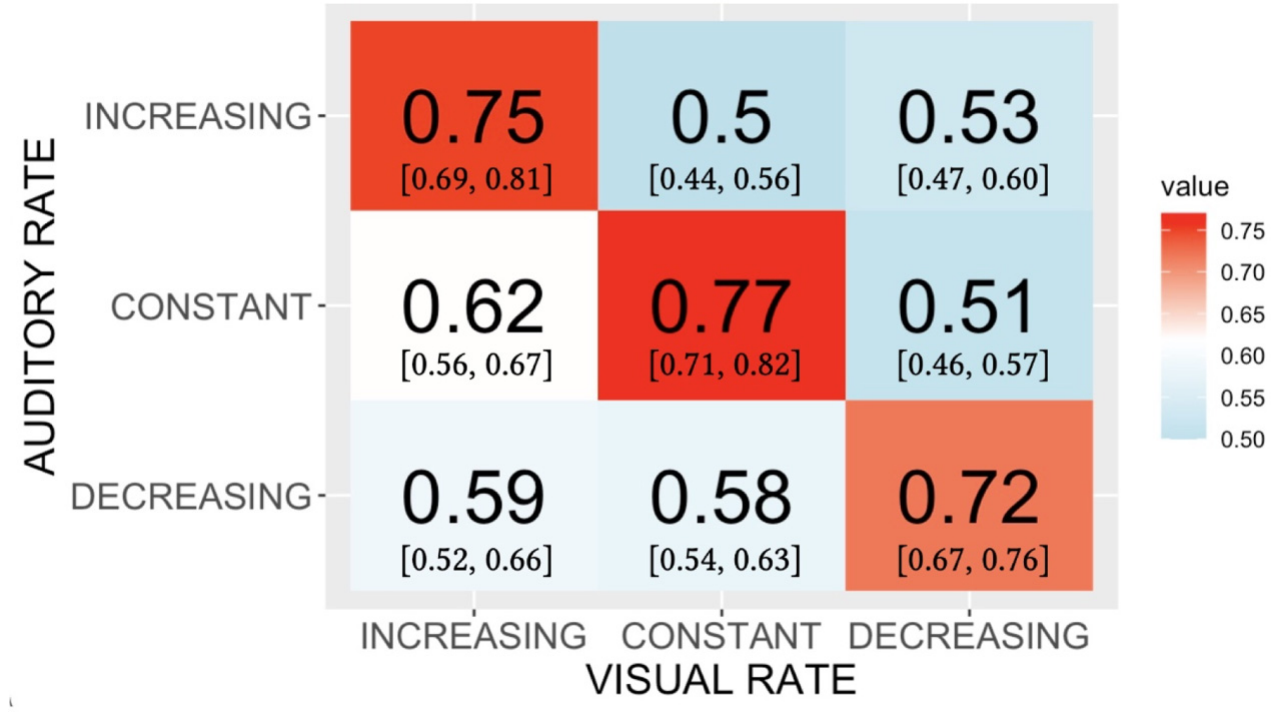
Phase B data showing values of correct responses only for various combinations of visual stimuli on the *x*-axis and auditory stimuli on the *y*-axis. The values of correct responses were extracted from the appropriate cells in Figure 4A to C. Note that this is not a confusion matrix, but an arrangement showing the proportion of correct responses for combination of visual stimuli and auditory stimuli.

To highlight another effect represented across Figure 4’s confusion matrices, we extracted values from cells representing correct responses, and aggregated them into a single matrix, Figure 5. Note that this is not a confusion matrix per se. Guided by previous results on audiovisual combination, we partitioned the nine auditory-visual combinations into two unequal sized sets: one comprised the three *Congruent* conditions represented along the negative diagonal of Figure 5, and the second set included the remaining six conditions located off the diagonal.

Overall mean accuracy for *Congruent* conditions was 0.747±0.029, while for *Incongruent* conditions it was = 0.555±0.032. A matched samples *t*-test confirmed the statistical reliability of the difference: *t*(20) = 74.34, *p* = 3.60×10^−8^. The difference of 0.19 between *Congruent* and *Incongruent* was as large or larger than differences reported previously, but with stimulus modulation rates that were invariant over time (Goldberg et al., 2015; Sun & Sekuler, 2021; Sun et al., 2017; Tai et al., 2022; Varghese et al., 2017). A two-way, repeated measures ANOVA provides a complementary perspective on the effect of shared modulation rate change. The ANOVA, summarized in Table 3, compared values along the negative diagonal of Figure 5 to corresponding values from Phase A of this study. The visual stimuli in this comparison were the same in both phases; only the accompanying auditory stimuli differed. Aggregated across conditions, mean accuracy was slightly, though not reliably, higher in Phase B than in Phase A: 0.747±0.029 and 0.745±0.035, respectively. The small difference between these two means (0.002) is an estimate of the benefit produced, in Phase B, by an auditory stimulus whose modulation was synchronized to that of the visual stimulus, *F*(1, 20) = 6.40, *p* = .060, $\eta_{p}^{2}$ =0.242.

**Table 3.** Summary of ANOVA comparing selected results from Phases A and B.

Effect	*df*s	*F*	*p*	$\eta_{p}^{2}$
Visual rate	1.97, 39.37	1.76	.371	0.081
Phase	1, 20	6.40	.060	0.242
Interaction	1.70, 33.94	1.42	.371	0.066

Corrected for multiple comparisons using Holm-Bonferroni method.

Results from Phase B showed that the sweep direction of a visual stimulus was judged more accurately when it was paired with a Congruent auditory sweep than when it was paired with an Incongruent auditory sweep (see Figure 5). To check that this outcome was not simply reducible to changes at a decision level, without actual interaction between responses to auditory and visual signals, we computed the signal detection measure $d^{\prime }$ (Macmillan & Creelman, 2005) for key pairs of conditions in Phase B. Specifically, we calculated two sets of $d^{\prime }$ values. One set of values compared Increasing and Constant visual stimuli for each of the three possible responses; the other set of three values compared Constant and Decreasing visual stimuli for the three possible responses. Within the signal detection framework, $d^{\prime }$ is an estimate of the separation between the distributions of sensory responses on which the response is based. In our case, one pair of distributions represents effects of Increasing and Constant visual stimuli, and the other pair of distributions represents effects of Constant and Decreasing visual stimuli. To calculate hits and false alarms needed for $d^{\prime }$, we treated Constant visual stimuli as the null (“noise”) condition and Increasing visual stimuli as “signal” for one set of calculations, and Decreasing visual stimuli as “signal” for the other. Table 4 shows the result. Note that of the three values of $d^{\prime }$ in each set, one represents a condition in which auditory and visual rate changes matched. For both of the matched auditory and visual rates, the absolute values of $d^{\prime }$ were smaller than for non-congruent conditions, that is, 0.93 < (1.43,1.45) and −1.11< (−1.36,−1.31). This implies that when the accompanying task-irrelevant auditory stimulus’ rate change matches that of the visual stimulus, the distributions underlying the decisions are closer together than when auditory and visual stimuli do not match in rate change. Thus, a match or mismatch between changes in visual and auditory rates affects sensitivity to the perceptual difference between those stimuli.

**Table 4.** Phase B data showing values of $d^{\prime }$ from selected pairwise comparisons.

	Visual Increasing	Visual Constant
Auditory stimuli	versus Constant	versus Decreasing
Increasing	0.93	−1.36
Constant	1.43	−1.31
Decreasing	1.45	−1.11

Table 1 and Figure 4 show that the accuracy of categorizing visual rate modulation varied with the accompanying auditory rate change. Some previous studies showed that change in stimulus appearance can be induced by task-irrelevant stimuli (e.g., Chen et al., 2019; Huang et al., 2009). Extrapolating from such results, we decided to determine whether the direction of auditory rate change promoted a perceptual change in the visual rate, a change in which the representation of a Constant visual rate would be attracted toward the auditory change in rate. For example, would a Decreasing task-irrelevant auditory rate cause subjects to misjudge the task-relevant Constant visual stimulus as decreasing, and would an Increasing auditory rate promote erroneous judgments that the Constant visual rate increased? Such effects, if found, would be akin to previously reported perceptual capture effects produced by task-irrelevant, static spatial stimuli (Huang & Sekuler, 2010; Makovski et al., 2010).

To answer this question, we partitioned the 419 relevant trials into a 2×2 table according to stimulus type and response choice (Table 2). A $\chi^{2}$ test of independence showed a statistically significant effect of the relation between the directional change in auditory rate and subjects’ judgments of the Constant visual modulation, $\chi^{2}$(1,419) = 115.07, *p*<.00001. Specifically, when the sound’s modulation was Increasing, subjects were 3.9× more likely to judge the accompanying disc’s modulation rate as increasing rather than decreasing; and when the auditory modulation rate was Decreasing, subjects were 2.7× more likely to respond that the visual modulation rate had also decreased than to respond that it had increased. Overall, on 77% of test trials judgments of the Constant rate visual stimulus shifted toward the direction—increasing or decreasing—in which the rate of the auditory stimulus was changing.

## Discussion

Results from Phase B of our study confirm what previous, related experiments had shown: a task-irrelevant, temporally modulating auditory stimulus induces errors in the judgment of a concurrent visual stimulus. This result is most readily apparent in the difference of about 19% between mean accuracy in Congruent conditions and mean accuracy in Incongruent conditions (Figure 5). That result generalizes from previous studies with stimuli whose modulation frequency was Constant, to our conditions in which modulation frequency can vary over time. However, the difference between results from the Congruent and Incongruent conditions in our experiment was nearly entirely caused by reduced accuracy when visual and auditory rate changes differed from one another, relative to the control Constant condition. Surprisingly, relative to the control, we found essentially no gain in accuracy when visual and auditory rate changes matched. This result differs from what previous studies demonstrated (Sun & Sekuler, 2021; Sun et al., 2017; Varghese et al., 2017; Zhou, 2019). It is hard to draw any firm conclusion about the origin(s) of this discrepancy, although a possible contribution from the dynamics of our stimuli cannot be dismissed out of hand.

Information generated by previous related studies was limited by reliance on binary judgments; their results merely showed when errors were made and their proportions. This limited, binary-decision landscape made them mute about the character of the errors. To circumvent this limitation, our study adopted a mutli-alternative decision-making framework with three stimulus classes and a corresponding number of response choices. This allowed us to make inferences about the character of subjects’ errors. The analysis summarized in Table 2 suggested that the dynamics of task-irrelevant auditory stimuli draw subjects’ judgments of concurrent visual stimuli toward their own direction of change in rate of oscillation, Increasing or Decreasing. Specifically, on slightly more than three-quarters of the relevant trials, misjudgments were in the direction—rate increasing or decreasing—of the task-irrelevant stimulus. As remarked earlier, this result resembles the auditory driving reported by Shipley (1964); it also bears some resemblance to attractor effects between static stimuli within one modality (Huang & Sekuler, 2010; Makovski et al., 2010).

Information generated by our multiple-alternative psychophysical method is a step up from merely knowing only that an error was made, but any additional information is silent about the error’s magnitude. Specifically, although we know the existence and direction of the error, we do not know the perceptual similarity of the misperceived stimulus to the inducing stimulus. Specifically, we cannot tell whether the misperception was small, just barely enough to bias perception slightly toward the inducing stimulus, or whether it was large enough to make the perceived stimulus very similar to or even indistinguishable from the inducing stimulus. An approach that might have greater information yield would supplement identification responses with subjects’ confidence in those identifications. However, the usefulness of this approach requires an assumption that confidence judgments are at least monotonically related to the distance between the underlying, continuous sensory evidence and the discrete response criteria subjects used (Fetsch et al., 2014; Mamassian, 2020). A very different method would abandon categorical judgments, even multi-alternative category judgments with or without confidence judgments, and, instead, substitute a response modality like the method of adjustment (Huang & Sekuler, 2010) or a continuous matching procedure (Fougnie et al., 2012; Motala et al., 2018; Shipley, 1964). These measurement methods afford more granularity than categorical responses do. For example, Shipley showed a nearly linear, one-to-one relationship up to ∼10 Hz between a change in auditory rate and the visual rate subjects deemed to match that changed rate. Although experiments like Shipley’s are commonly assumed to yield direct reflections of perception, guaranteeing that they reflect nothing whatever in addition to perception is challenging (Brindley, 1970; Morgan et al., 2013). After all, like our own experiment, a lot of research in perception does not focus on detection, discrimination, or metamerism, but on subjects’ assessment of stimulus appearance, that is, phenomenology (Alais & Burr, 2004).

Our study raises a number of questions that future work should address. For example, what stimulus information allows subjects to categorize stimuli according to the rate at which modulation varies over time? More specifically, what stimulus-based computations feed into the decision process? Two recent studies offer hints at possible answers. In one, Villalonga et al. (2020) tested subjects’ ability to discriminate between pulsatile stimuli (vibrotactile, visual, and combined vibrotactile and visual) that differed in mean rate and in temporal stochasticity. Accuracy was modeled by assuming that subjects based responses on information accumulated over all the intervals separating successive pulses, with particular weight given to intervals early in a sequence. In another recent study, Espinoza-Monroy & de Lafuente (2021) asked subjects to discriminate between regular and time-varying visual, auditory, or tactile pulsatile stimuli. Performance was modeled by assuming that subjects accumulated the perceived time differences between successive pulses, with little or no leak of information over time. Adding an appropriate differencing operator might allow one of these models to account for performance in our task. Note, though, that both models depend upon accurate registration of onset times for successive pulsatile stimuli. Extending that approach to our own non-pulsatile, sinusoidally modulating stimuli would require reliable detection and accumulation of successive, corresponding peaks in the modulation (or some other distinctive features). That could work in principle, but it is not clear how precisely and reliably the necessary information could be extracted from a continuous, sinusoidally modulating stimulus.

Any attempt to understand the basis for our subjects’ decision-making would benefit from a fuller exploration of stimulus properties. For one thing, our stimuli were limited to just a single rate of sweep, 21% sec${}^{-1}$, and that sweep was centered around only one modulation frequency, 5 Hz. Preliminary testing showed that sweep rate mattered, but did not constitute anything like a parametric exploration of that stimulus parameter. Among other things, knowing more about modulation rate and frequency could allow comparisons with stimuli whose rate of modulation did not vary over time. Additionally, by forcing subjects to delay responding until the entire two-second stimulus presentation had ended, we do not know at what rate decision-critical information was accumulating for different stimuli or when accumulation reached a level needed for a decision (Varghese et al., 2017). Such questions might be answered by allowing subjects to respond without waiting for a fixed observation period.

A second set of unanswered questions relates to the possible loci and mechanism(s) for the audiovisual interactions that produced the misperceptions in Phase B of our experiment. Using various tasks and measures, such as neurophysiology, electroencephalography and functional magnetic resonance imaging, many studies have examined audiovisual interaction. Their results have implicated multiple sites in the brain, subcortical structures (Meredith et al., 1987; Rowland & Stein, 2014) as well as multiple areas of the cerebral cortex (van Atteveldt et al., 2014; Bischoff et al., 2007; Calvert et al., 1997). Although sound can affect primary visual cortex (Murray et al., 2016; Shams et al., 2001), it is unclear how such cortical effects can explain how the perceived temporal dynamics in one modality could be altered by a concurrent dynamic stimulus in another modality (Motala et al., 2018). Although many cortical sensory areas have been shown to respond to inputs from more than just one modality, that fact does not establish how specific stimulus properties, such as frequency and frequency sweep direction, are represented in those multimodal responses. In one step toward an answer, a recent study exploited repetition suppression in functional magnetic resonance imaging to reveal some co-localization of multimodal frequency selective responses to auditory and tactile stimuli (Rahman et al., 2020). It remains to be seen whether those results will generalize to frequency sweep stimuli or to multisensory combinations other than auditory and tactile.
